# The Value of ^18^F-FDG PET/CT in Diagnosing Pancreatic Lesions: Comparison With CA19-9, Enhanced CT or Enhanced MR

**DOI:** 10.3389/fmed.2021.668697

**Published:** 2021-10-08

**Authors:** Shengyun Huang, Huanhuan Chong, Xun Sun, Zhijian Wu, Qing Jia, Yongxue Zhang, Xiaoli Lan

**Affiliations:** ^1^Department of Nuclear Medicine, Union Hospital, Tongji Medical College, Huazhong University of Science and Technology, Wuhan, China; ^2^Department of Nuclear Medicine, National Cancer Center/National Clinical Research Center for Cancer/Cancer Hospital and Shenzhen Hospital, Chinese Academy of Medical Sciences and Peking Union Medical College, Shenzhen, China; ^3^Hubei Key Laboratory of Molecular Imaging, Wuhan, China; ^4^Department of Radiology, Ruijin Hospital, Shanghai Jiao Tong University School of Medicine, Shanghai, China

**Keywords:** ^18^F-FDG PET/CT, pancreatic lesions, serum CA19-9, enhanced CT, enhanced MR, diagnosis

## Abstract

**Objective:** To investigate the value of ^18^F-FDG PET/CT in diagnosing pancreatic lesions, and compare it with CA19-9, contrast-enhanced CT (CECT), and contrast-enhanced MRI (CEMR).

**Methods:** Cases of patients with suspected pancreatic lesions examined between January 1, 2011 and June 30, 2017 were retrospectively analyzed. CA19-9, CECT and CEMR within 2 weeks of PET/CT were evaluated. We compared the diagnostic efficacy of PET/CT with CA19-9, CECT and CEMR as well as combined tests.

**Results:** A total of 467 cases were examined in this study, including 293 males and 174 females, with an average age of 57.79 ± 12.68 y (16–95 y). Cases in the malignant group (*n* = 248) had significantly higher SUVmax (7.34 ± 4.17 vs. 1.70 ± 2.68, *P* < 0.001) and CA19-9 (663.21 ± 531.98 vs. 87.80 ± 218.47, *P* < 0.001) than those in the benign group (*n* = 219). The sensitivity, specificity and accuracy of PET/CT were 91.9, 96.3, and 94.0%, respectively. Those for CECT were 83.6, 77.8, 81.2%, respectively; and 91.2, 75.0, 81.7% were for CEMR. PET/CT corrected 14.7% (28/191) CECT diagnoses and 12.2% (10/82) CEMR diagnoses. Although the diagnostic efficiency of CA19-9 was acceptable (80.0, 69.0, 74.9% respectively), the joint application of PET/CT and CA19-9 could significantly enhance the diagnostic efficiency compared with PET/CT alone (sen 97.4 vs. 90.5%, *P* = 0.0003; spe 100.0 vs. 95.2%, *P* = 0.0047).

**Conclusions:** PET/CT has sensitivity similar to CECT, CEMR and significantly higher specificity and accuracy, helping reduce false diagnoses of morphological images. Combining PET/CT with CA19-9 could enhance diagnostic efficiency.

## Introduction

Pancreatic cancer is one of the most fatal tumors in the world with a median survival time of merely 3–6 months. Its incidence and mortality have continued to rise in the past decade (1, 2). Only surgery is curative for patients with pancreatic cancer. Early symptoms of pancreatic cancer (including epigastric and back pain, jaundice, and weight loss) are insidious and non-specific (3). About 60% of pancreatic cancer patients have distant metastases at the time of diagnosis (4). The main challenge in clinical practice for patients with pancreatic cancer is to accurately distinguish malignant lesions from benign ones in early evaluation.

Carbohydrate antigen 19-9 (CA19-9) is the most commonly used tumor marker in the management of pancreatic cancer. But it is frequent false-positive in pancreatitis, cirrhosis, and in other gastrointestinal cancers such as colorectal cancer and cholangiocarcinoma carcinoma, and inevitably false-negative in Lewis antigen-negative subpopulations (5). The standard imaging modality recommended by NCCN guidelines is contrast-enhanced multi-detector computed tomography (CECT) (6). Remarkable advances in CT technology have improved its ability to precisely assess local invasion of primary tumor. However, there are still limitations when suspected lesions are ambiguous on CT or when CECT cannot be obtained. Contrast-enhanced magnetic resonance imaging (CEMR) with superior soft tissue resolution and high sensitivity is particularly helpful in these situations. Yet both CECT and CEMR are still restricted to morphological portrait of tumor, leading to inaccurate diagnosis of certain patients.

The rapid development of positron emission tomography/computed tomography (PET/CT) since the 1990s has enabled a comprehensive assessment of both morphology and metabolic activity of lesions. Overexpression of glucose transporter-1 (GLUT-1) and increased glucose utilization are reported in pancreatic carcinoma (PC), making it possible to detect PC early with ^18^F-FDG PET (7). However, current guidelines (NCCN and ESMO) do not clearly define the role of PET/CT in pancreatic cancer (6, 8). This study compared PET/CT with other traditional tests (CA19-9, CECT, and CEMR) in the diagnosis of pancreatic lesions. We then further explored whether the diagnostic efficiency could be improved by combing different methods.

## Materials and Methods

### Patients

Cases of patients from January 1, 2011 to June 30, 2017 with suspected pancreatic lesions were retrospectively analyzed in our PET center. Inclusion criteria were as follows: (1) no pathological diagnosis, or any anticancer therapy including surgery, radiotherapy, or chemotherapy before any examination; (2) serum CA19-9, CECT or CEMR performed within 2 weeks of PET/CT if available; (3) complete medical history and follow-up data (>6 months). Exclusion criteria were: (1) blood glucose >11 mmol/L before injection of ^18^F-FDG; (2) other malignant tumors in addition to the pancreatic lesions confirmed either before or after PET/CT scan; (3) pancreatic neuroendocrine tumors/cancers (NETs/NECs). The reference standard for diagnosis was based on histology (either biopsy or surgery) and/or clinical outcome assessment. This study was approved by the Institutional Review Board of Union Hospital, Tongji Medical College, Huazhong University of Science and Technology.

### PET/CT Protocol

All patients were requested to fast for at least 4–6 h before the PET/CT examination, and their blood glucose levels were ≤ 11 mmol/L before ^18^F-FDG injection. Patients were intravenously injected with 3.7–5.55 MBq/kg ^18^F-FDG of ≥95% radiochemical purity synthesized by a cyclotron (GE Minitracer®, GE Healthcare, Milwaukee WI, USA) and synthesizer (TracerLab MX-FDG®, GE). Patients were required to rest in a quiet, dark room for ~60 min and drink 300–500 mL of water before examination. After emptying the bladder, patients were scanned (Discovery LS® or VCT PET/CT/CT®, GE Healthcare, Milwaukee WI, USA). A CT scout view was performed followed by a low-dose CT scan (120 kV, 100 mA, and 3.75 mm slice thickness). Whole-body PET scanning was then performed immediately from the top of skull to the upper thighs at six to eight bed positions (2–3 min per bed position). Delayed abdominal scan (2–3 h after injection) was occasionally required for lesions that were inconspicuous or equivocal in early phase. 3D PET images were reconstructed by an iterative algorithm, using the CT image data for attenuation correction and then transferred to a workstation (Xeleris®, GE).

### Image Analysis

PET/CT, CECT, CEMR and other clinical data were retrieved from hospital databases. Diagnoses of pancreatic lesions were independently made by at least two experienced nuclear medicine or radiology physicians. Final consensus was reached after comprehensive image analysis. Visual and semi-quantitative methodology (maximum standardized uptake value, SUVmax) were applied to PET/CT analysis. Retention index (RI) (9, 10) were used for dual-phase PET/CT. The retention index (RI) was calculated as follows:


$$\begin{array}{l} \text{RI}=\frac{\text{SUV2-SUV1}}{\text{SUV1}}\times \text{100} \end{array}$$

In general, lesions with focal ^18^F-FDG uptake (SUVmax ≥ 2.5 or exceeding normal pancreas uptake) or significantly increased uptake on delay scan, evidence of local invasion, or distant metastasis was considered as suspicious for malignancy. CECT and CEMR diagnosis of pancreatic lesions were assessed according to the NCCN guidelines and radiology reporting template (6, 11).

### Statistical Analysis

The data are presented as mean ± standard deviation. Differences in SUVmax and serum CA19-9 between malignant and benign diagnoses were compared with an independent-samples *t*-test. The optimal cut-off points with maximum Youden Index were calculated by receiver operating characteristic (ROC) analysis. Sensitivity, specificity, accuracy, positive predictive value (PPV), and negative predictive value (NPV) of PET/CT, CA19-9, CECT, and CEMR were calculated. The results from different tests were also analyzed in parallel and in serial, to determine whether combinations of tests might give better performance. For parallel tests, results are defined to be positive as long as any one is positive, or negative when both are negative. For serial tests, positive results are considered only when both tests are positive. The diagnostic efficacies of the different methods were compared using the McNemar's chi-squared test. All statistics were generated using statistical packages from R software (www.rproject.org, Version 3.4; “pROC” and “ggplot2”). A two-tailed test with *P-*value <0.05 was considered statistically significant.

## Results

### General Characteristics

General characteristics of all 467 patients are detailed in Table 1. The average age was 57.79 ± 12.68 y (range, 16–95 y). Patients ≥ 70 y were 2.37 times more likely to have pancreatic cancer than those <70 y (*P* < 0.001). Cases were divided into malignant (*n* = 248) and benign groups (*n* = 219) by histology (either biopsy or operation) in 142 cases, and clinical outcome assessment (at least 6 months follow-up) in 325 cases. Follow-up was carried out until December 2017. Median follow-up time was 25.7 months (range, 6.1–76.1 months). For the 142 cases who had histology results, 91 were malignant and 51 were benign (Table 2).

**Table 1:** General characteristics of cases.

	**Malignant**	**Benign**	**All cases (%)**	**OR**
				***P-*value**
Total	248	219	467	
Sex				OR = 1.27
Male	162	131	293 (62.7)	*P* = 0.25
Female	86	88	174 (37.3)	
Age			57.79 ± 12.68 y	OR = 2.37
≥70 y	64	28	9 2 (19.7)	*P* < 0.001
<70 y	184	191	375 (80.3)	
**Location of lesions on pancreas**				
Head/neck	102	77	179 (38.3)	
Body/tail	133	71	204 (43.7)	
Whole pancreas	7	42	49 (10.5)	
Diffused/multiple lesions	6	11	17 (3.6)	
Not obvious	0	18	18 (3.9)	
**Clinical stage**				
I/II	98	———		
III/IV	150	———		

**Table 2:** Histology results of 142 patients.

**Malignant cases**	**91**
Pancreatic ductal adenocarcinoma (PDAC)	9
Serous/Mucinous cystadenocarcinoma	2
Intraductal papillary mucinous neoplasm (IPMN) with atypical hyperplasia	2
Tubular adenocarcinoma	10
Adenocarcinoma (unspecified)	44
Solid-pseudopapillary carcinoma (SPT)	2
IPMN-related invasive carcinoma - ductal adenocarcinoma with neuroendocrine microadenomas	1
Undifferentiated carcinoma	1
Found multiple metastasis intraoperatively	18
Limited sample^*^	2
**Benign cases**	**51**
**Pancreatitis**	
Acute pancreatitis	5
Chronic pancreatitis	14
Chronic lymphoplasmacytic pancreatitis	1
Pancreatic pseudocyst	2
Autoimmune pancreatitis	3
**Pancreatic benign tumor**	
Serous/Mucinous cystadenoma	7
Giant Pancreatic lipoma	1
Pancreatic tuberculosis	2
Normal pancreatic tissue/no obvious lesion was observed^**^	16

* *Two patients without positive finding due to limited sample of biopsy, but then diagnosed as pancreatic cancer during clinical follow-up*.

** *Sixteen patients without significant abnormal finding on biopsy or multiple imaging's, and were cured after treatment*.

### The Diagnostic Efficacy of PET/CT and Derived Parameters

**Figure 1:**
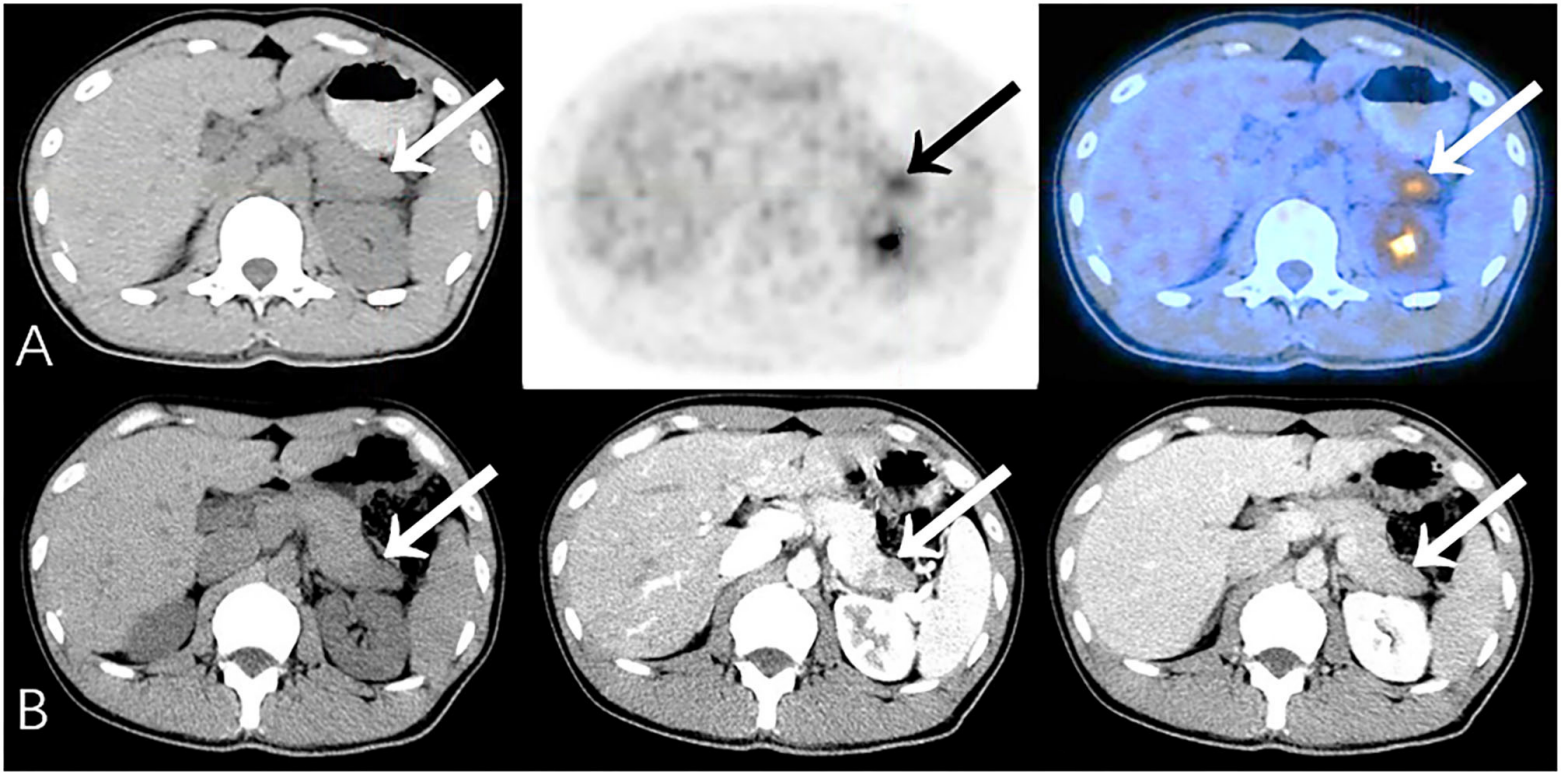
Representative patient on PET/CT (False-Positive) compared to CECT (True-Negative): A 34-year-old male had abdominal pain for 2 months, worsening in the last 10 days. Serum CA19-9 was normal (34.1 U/mL). **(A)** PET/CT showed significantly increased ^18^F-FDG uptake area in the tail of the pancreas (SUVmax 5.5). The edge of the lesion was indistinct and seemed to be closely related to the stomach wall; it was considered to be malignant. **(B)** Density of the lesion was relatively low at every phase. Fat spaces around the pancreas were clear on CECT (benefits from the higher resolution), suggesting chronic pancreatitis. Patient underwent distal pancreatectomy, and histopathology revealed chronic pancreatitis with small abscesses. No special complaints during follow-up (>23 months).

In general, the sensitivity, specificity, accuracy, PPV and NPV of PET/CT were 91.9, 96.3, 94.0, 96.6, and 91.3%, respectively. For those cases misdiagnosed by PET/CT (Table 3), pancreatitis and tuberculosis were the most important false-positive findings on PET/CT (Figure 1), while medium-/well-differentiated pancreatic cancers tend to be negative on PET/CT.

**Table 3:** Misdiagnosed cases of PET/CT.

**PET/CT false-positive (FP)**	**8**
**Pancreatitis**	
Chronic pancreatitis	1
Chronic lymphoplasmacytic pancreatitis	1
Autoimmune pancreatitis	2
Inflammatory lesions	1
**Pancreatic tuberculosis**	2
Benign mass^*^	1
**PET/CT false-negative (FN)**	**20**
**By histology**	
Well-differentiated adenocarcinoma	1
Medium-well-differentiated adenocarcinoma	1
Medium-well-differentiated tubular adenocarcinoma	1
Medium differentiated ductal adenocarcinoma	2
Poor differentiated mucinous cystadenocarcinoma	1
Adenocarcinoma (unspecified)	3
Solid-pseudopapillary carcinoma (SPT)	1
Multiple metastasis found during operation	3
**By clinical follow-up**	7

* *1 case refuse to carry out biopsy and was clinically diagnosed as benign by multi-disciplinary consultation, cured after supportive treatment (follow-up >16 months)*.

**Figure 2:**
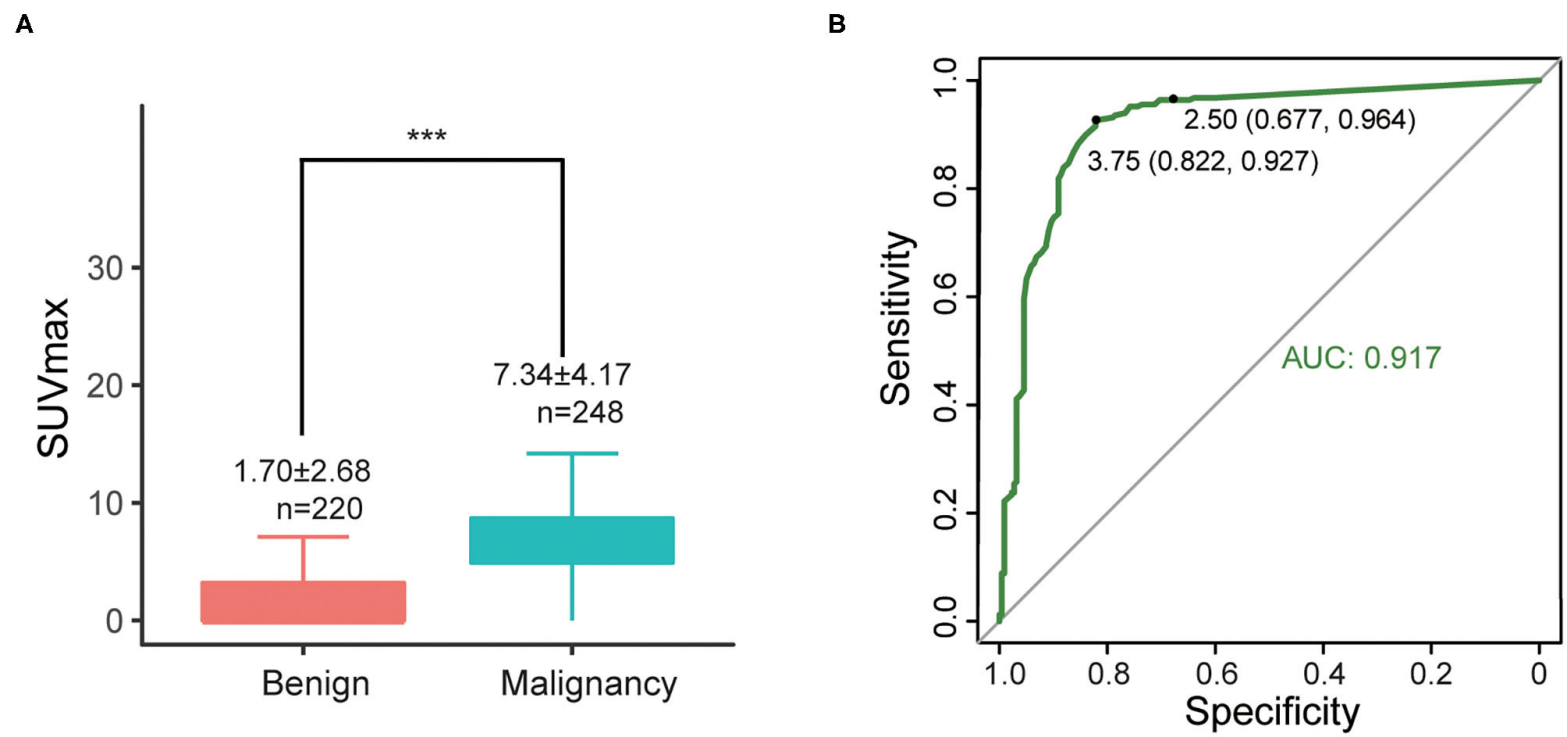
**(A)** Differences of SUVmax between malignant and benign groups, ***P < 0.001; **(B)** ROC curve of SUVmax (all cases).

SUVmax of primary lesions were measured for all 467 cases. SUVmax in the malignant group were significantly higher than benign group (7.34 ± 4.17 vs. 1.70 ± 2.68, *P* < 0.001) (Figure 2A). According to ROC curves (Figure 2B), the areas under the curve (AUC) of SUVmax were 0.917. The best diagnostic performances were achieved when the optimal cut-offs were set at SUVmax = 3.75 (with sensitivity of 92.7% and specificity of 82.2%) compared with the conventional standard cut-off 2.5 (with sensitivity of 96.4% and specificity of 67.7%).

**Figure 3:**
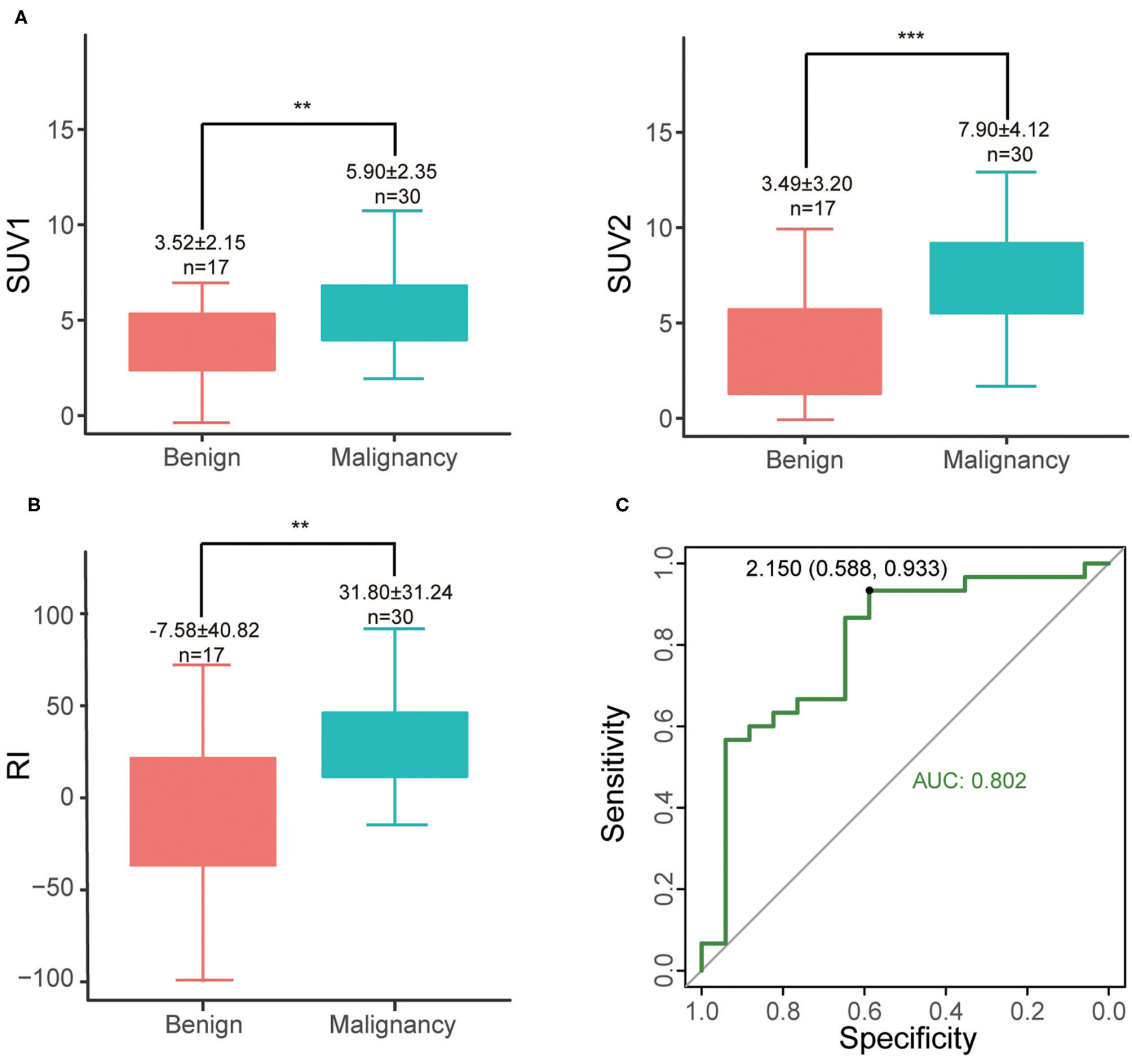
**(A)** Differences of SUV1 and SUV2 between malignant and benign groups, **P < 0.01; ***P < 0.001; **(B)** RI of different groups; **(C)** ROC curve of RI.

Among 47 patients (47/467, 10.1%) examined with delayed abdominal scan, cases in the malignant group tend to have higher SUV1 (5.90 ± 2.35 vs. 3.52 ± 2.15, *P* = 0.0012), SUV2 (7.90 ± 4.12 vs. 3.49 ± 3.20, *P* < 0.001) and RI (31.80 ± 31.25 vs. −7.58 ± 40.82, *P* = 0.0019) than those in the benign group (Figures 3A,B). The optimal cut-off was RI = 2.15 (with sensitivity of 93.3% and specificity of 58.8%) (Figure 3C). Increased SUV2 (or RI > 0) is noted in most of malignant lesions (28/30, sen 93.3%), except for 2 malignant cases (1 medium differentiated adenocarcinoma and 1 unspecified adenocarcinoma). As for benign cases, 3 maintained stationary, 7 had decreased SUVmax (10/17, spe 58.8%), and the rest 7 cases revealed an increase in SUVmax (7/17, 41.2%) including 1 chronic lymphoplasmacytic pancreatitis, 1 autoimmune pancreatitis and 5 pancreatitis.

### Diagnostic Efficiency of PET/CT Compared With CA19-9

**Figure 4:**
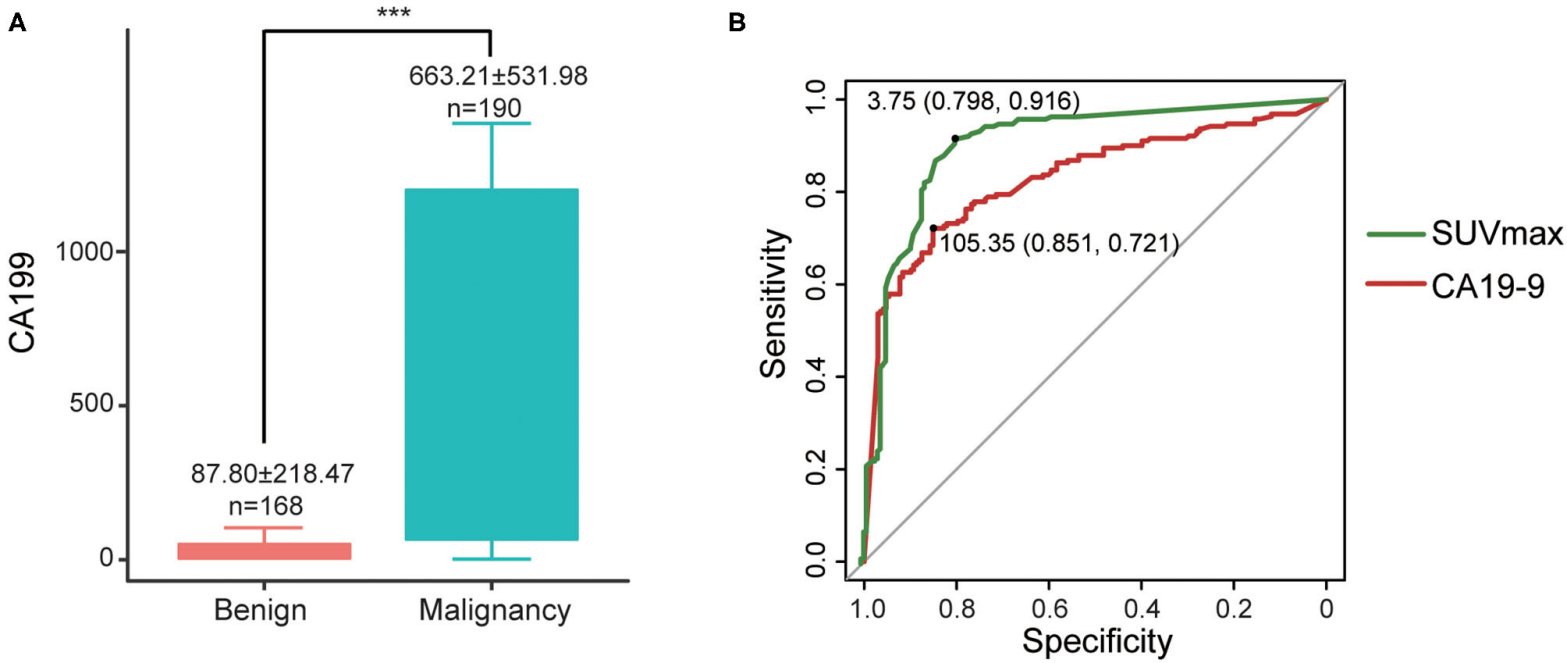
**(A)** Differences of CA19-9 between malignant and benign groups, ***P < 0.001; **(B)** ROC curves of CA19-9 and SUVmax.

Cases in the malignant group had significantly higher CA19-9 (663.21 ± 531.98 vs. 87.80 ± 218.47, *P* < 0.001) than those in the benign group (Figure 4A). The sensitivity, specificity, and accuracy of CA19-9 alone were 80.0, 69.0, and 74.9%, respectively.

For the 358 cases which underwent both PET/CT and serum CA19-9 within 2 weeks (including only the latest CA19-9 result from PET/CT if repeatedly assessed), we compared their diagnostic efficiency by ROC curves. The areas under the curves (AUCs) of SUVmax and serum CA19-9 were 0.90 and 0.831, respectively, indicating that the diagnostic efficiency of SUVmax is higher than that of serum CA19-9. The best diagnostic performances were achieved when the optimal cut-offs were set at 3.75 for SUVmax (sen 91.6%, spe 79.8%) and 105.35 for CA19-9 (sen 72.1%, spe 85.1%) (Figure 4B).

Diagnostic efficiencies were significantly improved when combined PET/CT with CA19-9 compared to PET/CT alone (parallel test: sen 97.4 vs. 90.5%, *P* = 0.0003; serial test: spe 100.0 vs. 95.2%, *P* = 0.0047). Moreover, the high NPV of parallel test (95.6%) indicates that negative results of both CA19-9 and PET/CT decrease the odds of a malignant diagnosis. Similarly, positive results of both two tests increase malignance possibility with a PPV of 100.0% for serial test (Table 4).

**Table 4:** Diagnostic efficiency of PET/CT compared with other tests.

	**Sensitivity**	**Specificity**	**Accuracy**	**PPV**	**NPV**
PET/CT (*n* = 467)	91.9%	96.3%	94.0%	96.6%	91.3%
**PET/CT**^**Δ**^ **with CA19-9 (*****n*** **=** **358)**					
PET/CT	90.5%	95.2%	92.7%	95.6%	89.9%
CA19-9	80.0%^**^	69.0%^***^	74.9%	74.5%	75.3%
PET/CT//CA19-9	97.4%^***^	64.3%^***^	81.8%	75.5%	95.6%
PET/CT + CA19-9	73.2%^***^	100.0%^**^	85.8%	100.0%	76.7%
**CECT**^**Δ**^ **with PET/CT (*****n*** **=** **191)**					
CECT	83.6%	77.8%	81.2%	83.6%	77.8%
PET/CT	89.1%^N.S.^	96.3%^***^	92.1%	97.0%	86.7%
PET/CT//CECT	94.5%^***^	76.5%^N.S.^	86.9%	84.6%	91.2%
PET/CT + CECT	78.2%^*^	97.5%^***^	86.4%	97.7%	76.7%
**CEMR**^**Δ**^ **with PET/CT (*****n*** **=** **82)**					
CEMR	91.2%	75.0%	81.7%	72.1%	92.3%
PET/CT	88.2%^N.S^	93.8%^**^	91.5%	90.9%	91.8%
PET/CT // CEMR	94.1%^N.S.^	75.0%	82.9%	72.7%	94.7%
PET/CT + CEMR	85.3%^N.S.^	93.8%^**^	90.2%	90.6%	86.5%

*Statistical significance compared with test**Δ***:

*** *P <0.001*;

** *P <0.01*;

* *P <0.05; N.S. = not significant*.

*When equal, McNemar test is inapplicable*.

### Diagnostic Efficiency of PET/CT Compared With CECT or CEMR

Among 467 cases, 191 underwent CECT, while 82 cases had CEMR. The sensitivity, specificity and accuracy of CECT were 83.6, 77.8, 81.2%, respectively. And those for CEMR were 91.2, 75.0, 81.7%, respectively.

PET/CT had similar sensitivity to CECT (89.1 vs. 83.6%, *P* = 0.16) and CEMR (88.2 vs. 91.2%, *P* = 0.56). But its specificity was significantly better than those of CECT and CEMR (96.3 vs. 77.8%, *P* < 0.001; 93.8 vs. 75.0%, *P* = 0.0027). The accuracy of PET/CT was over 90%.

**Figure 5:**
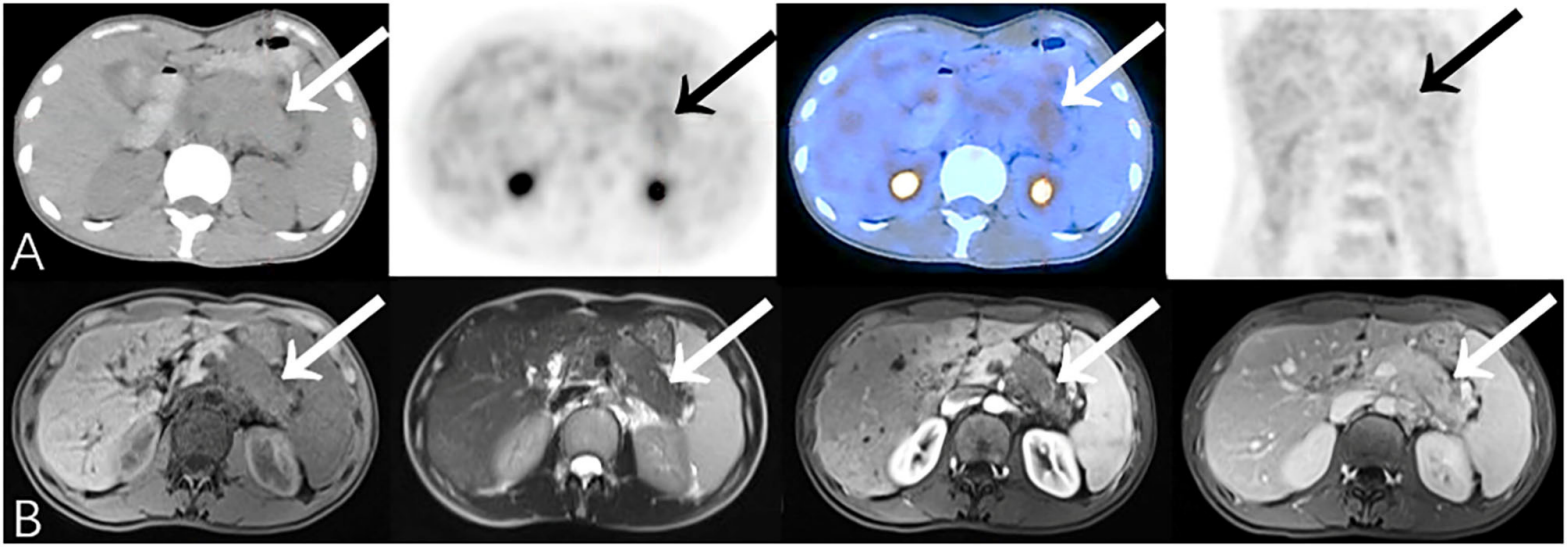
Representative patient on PET/CT (False-Negative) compared to CEMR (True-Positive): A 37-year-old female experienced epigastric pain for 4 months. Serum CA19-9 was elevated (259.4 U/mL). **(A)** The body and tail of pancreas were slightly swollen with slightly increased ^18^F-FDG uptake (SUVmax 3.1). There was insufficient evidence of malignancy on PET/CT especially when no other significant abnormality was identified on the rest of the body scan. **(B)** CEMR showed a low-signal area on T1WI with narrowing of the pancreatic duct in the body and tail of pancreas which has no significant density change on PET/CT due to relatively limited resolution. The lesion enclosed retroperitoneal vessels which was suggestive of pancreatic cancer. Biopsy result showed adenocarcinoma (differentiation unspecified). Patient died after 9.2 months.

Combined CECT with PET/CT can enhance diagnostic efficiencies compared to CECT alone (sen 94.5 vs. 83.6% for parallel test; spe 97.5 vs. 77.8% for serial test, both *P* < 0.001). Although CEMR was less specific (75.0%) for diagnosis, it seemed to perform better at detection and excluding malignant lesions with relatively high sensitivity (91.2%) and NPV (92.3%) (Figure 5). Furthermore, Combined CEMR with PET/CT can significantly improve specificity compared to CEMR alone (93.8 vs. 75.0%, *P* = 0.0027), but not better than PET/CT (Table 4).

**Figure 6:**
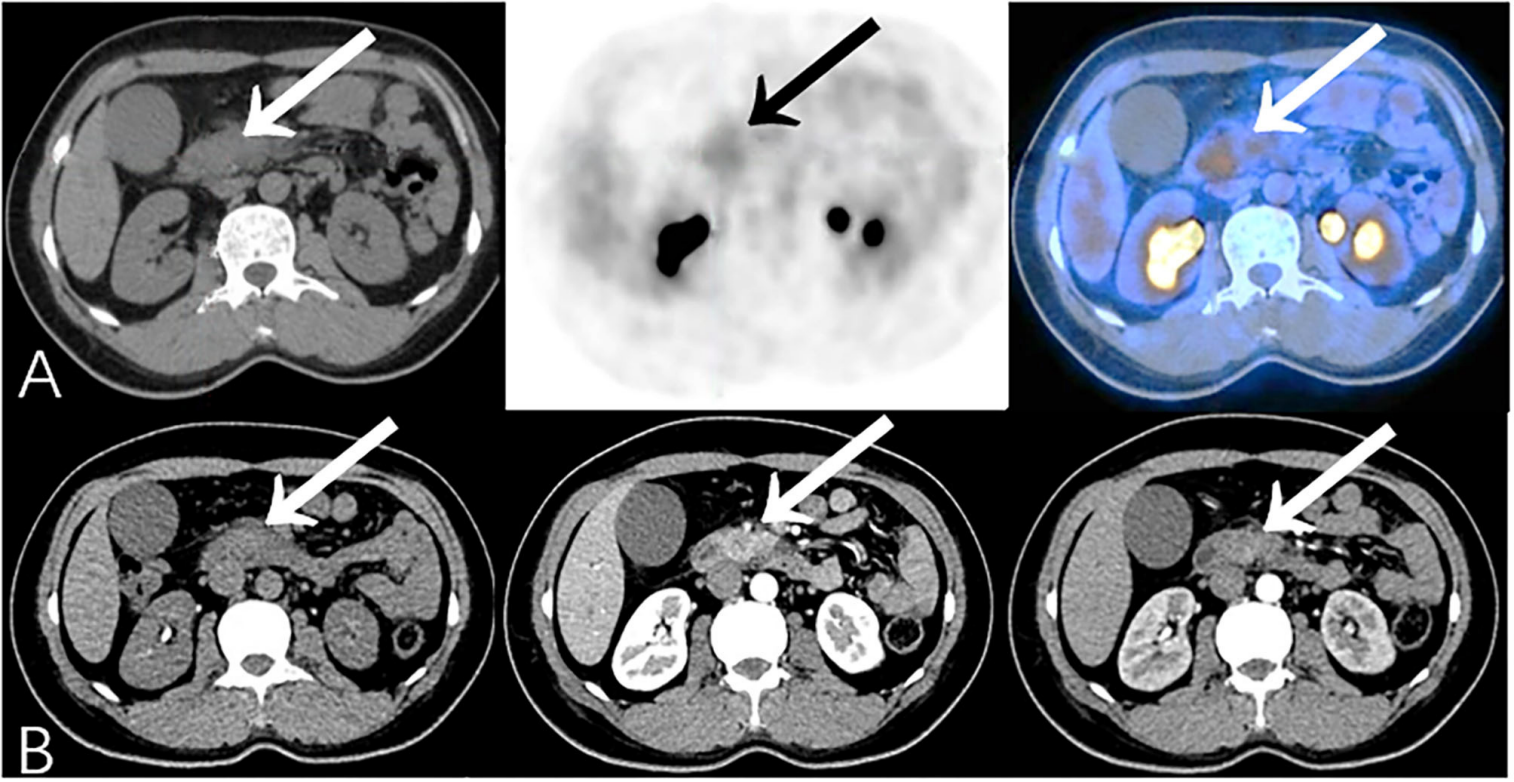
Additional findings on PET/CT (True-Positive) compared to CECT (False-Negative): A 45-year-old female experienced jaundice with abdominal pain for 2 months. Serum CA19-9 was elevated (926.7 U/ml). **(A)** PET/CT showed focal ^18^F-FDG uptake in the head of the pancreas (SUVmax 3.6). **(B)** CECT only showed obstruction, calcification, and the atrophy of pancreatic parenchyma subsequent to chronic pancreatitis. Patient then underwent pancreaticoduodenectomy, and histopathology revealed moderately-differentiated pancreatic ductal adenocarcinoma complicated with chronic pancreatitis.

**Figure 7:**
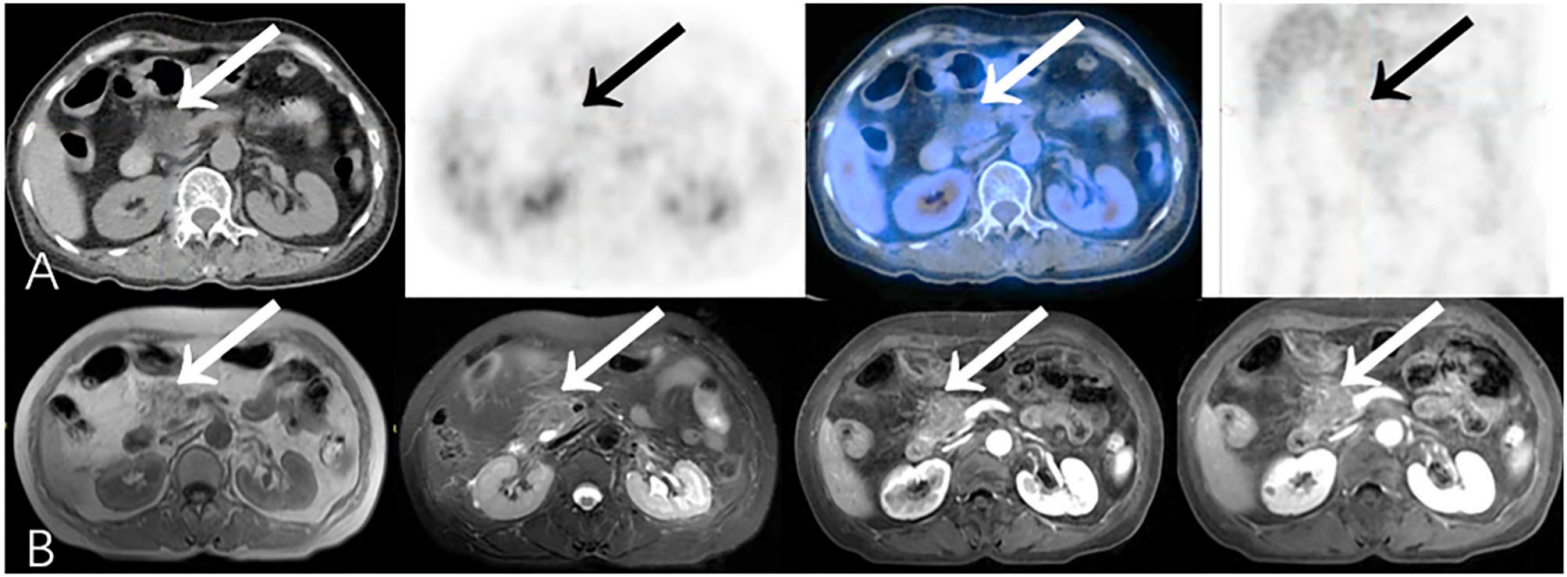
Representative patient on PET/CT (True-Negative) compared to CEMR (False-Positive): A 61-year-old female experienced abdominal pain, nausea and vomiting for 20 days. Serum CA19-9 was normal (12.2 U/ml). **(A)** PET/CT showed hypodense area without obvious increased focal ^18^F-FDG uptake (SUVmax 1.8) in head of pancreas, which was considered as benign lesion. **(B)** Lesion was inhomogeneously mildly enhanced on CEMR imaging, presumed more likely to be malignancy. Patient refused to undergo biopsy, and improved after symptomatic treatments. During long-term clinical follow-up (>16 months), patient had no complaints of special discomfort.

Some malignant lesions may lack typical malignant signs on morphology, which makes them harder to diagnose only by CT or MR. In our study, PET/CT corrected 14.7% (28/191) CECT diagnoses, identifying 12 malignant and 16 benign cases with the opposite diagnosis on CECT, and changed 12.2% (10/82) diagnoses by CEMR (Table 5). Additional metabolic information provided by PET/CT may lead to an improvement in the diagnostic accuracy of pancreatic lesions and in subsequent management of these patients (Figures 6, 7).

**Table 5:** Cross-tabulated diagnosis of PET/CT and CECT, CEMR.

**Diagnosis**	**Malignant**		**Benign**	
	**PET/CT positive**	**PET/CT negative**	**PET/CT positive**	**PET/CT negative**
CECT positive	86	6	2	16
CECT negative	12	6	1	62
CEMR positive	29	2	3	9
CEMR negative	1	2	0	36

### Compare Diagnostic Efficiencies of Other Combinations

CA19-9 could help improve the diagnostic efficiency combined with CECT (sen 95.8 vs. 83.2% for parallel test, *P* < 0.001; spe 97.0 vs. 83.3% for serial test, *P* = 0.0027) or CEMR (spe 97.6 vs. 78.0% for serial test, *P* = 0.0047) compared to the two imaging modalities alone. Given that sensitivity of CEMR is already pretty high, sensitivity of combined diagnosis was not significantly improved (sen 100 vs. 90.0% for parallel test, *P* = 0.083). Although combination of CECT and CEMR showed relatively higher sensitivity and specificity, it had no significant difference compared with CECT alone (sen 100.0 vs. 88.2% for parallel test, *P* = 0.157; spe 96.4 vs. 85.7% for serial test, *P* = 0.083), which may due to limited cases in this group (*n* = 45) (Table 6).

**Table 6:** Diagnostic efficiencies of other combinations.

	**Sensitivity**	**Specificity**	**Accuracy**	**PPV**	**NPV**
**CECT**^**Δ**^ **with CA19-9 (*****n*** **=** **161)**					
CECT	83.2%	83.3%	83.2%	87.8%	77.5%
CA19-9	74.7%^N.S.^	62.1%^*^	69.6%	74.0%	63.1%
CECT//CA19-9	95.8%^***^	48.5%^***^	76.4%	72.8%	88.9%
CECT + CA19-9	62.1%^***^	97.0%^**^	76.4%	96.7%	64.0%
**CEMR**^**Δ**^ **with CA19-9 (*****n*** **=** **71)**					
CEMR	90.0%	78.0%	83.1%	75.0%	91.4%
CA19-9	86.7%^N.S.^	58.5%^N.S.^	70.4%	60.5%	85.7%
CEMR//CA19-9	100.0%^N.S.^	39.0%^***^	64.8%	54.5%	100.0%
CEMR + CA19-9	76.7%^*^	97.6%^**^	88.7%	95.8%	85.1%
**CECT**^**Δ**^ **with CEMR (*****n*** **=** **45)**					
CECT	88.2%	85.7%	86.7%	78.9%	92.3%
CEMR	100.0%^N.S.^	85.7%^N.S.^	91.1%	81.0%	100.0%
CECT//CEMR	100.0%^N.S.^	75.0%^N.S.^	84.4%	70.8%	100.0%
CECT + CEMR	88.2%	96.4%^N.S.^	93.3%	93.8%	93.1%

*Statistical significance compared with test^Δ^*:

*** *P <0.001*;

** *P <0.01*;

* *P <0.05; N.S. = not significant*.

*When equal, McNemar test is inapplicable*.

## Discussion

In our study, we retrospectively evaluated a relatively large group of cases with suspected pancreatic lesions examined by PET/CT, CECT, CEMR and CA19-9. We compared not only the independent diagnostic value of ^18^F-FDG PET/CT, CA19-9, CECT and CEMR, but also the diagnostic efficacy of different combined tests. Our results indicate that ^18^F-FDG PET/CT performs better than the other three examinations in diagnosis of pancreatic lesions, especially in terms of specificity and accuracy. Moreover, the joint application of PET/CT with other methods could enhance the diagnostic efficiency.

PET/CT has made distinct progress for the diagnosis of tumors since the 1990s. As previous reported, the diagnostic efficacy of ^18^F-FDG PET/CT in pancreatic lesions varies from 85 to 100% in sensitivity, 61 to 94% in specificity, and 84 to 95% in accuracy (12–17). One of the reasons for the wide variation was the limited sample size. The heterogeneity of imaging technology between early studies may also affect the results. According to our research, ^18^F-FDG PET/CT has rather high sensitivity, specificity, and accuracy (91.9, 96.3, and 94.0%, respectively). Additional metabolic information provided by PET/CT may lead to an improvement in the diagnostic efficacy. Besides, PET/CT has larger field-of-view, which can help to detect metastasis and confirm the malignant diagnosis. However, tumor heterogeneity may contribute to inconsistent performance of PET/CT. It is important to note that medium- or well-differentiated pancreatic cancers tend to be negative on PET/CT. Higher tumor uptake, evaluated by the tumor SUVmax or the tumor-to-liver SUVmax ratio (SUVmax T/L), seems correlate to increased Ki67 and worse prognosis (18, 19). The overexpression of GLUT-1 play an important role in FDG uptake and accumulation in pancreatic cancer, which was reported to have positive correlations to SUV and histological grade though controversial (20–22). PET/CT may also have a role in the diagnosis of malignant cystic neoplasms (17). It is reported that malignant high-grade IPMN have significantly higher SUVmax (3.5 ± 1.4, *n* = 9) than the low-grade IPMN group (1.9 ± 1.1, *n* = 9), but with overlapped range between groups (23). Pancreatic solid pseudopapillary tumor (SPT) is also associated with increased FDG uptake (5.9 ± 5.7, *n* = 10) which was similar to PDAC (5.8 ± 2.7, *n* = 46) (24). However, PET/CT may be false-positive in cases with pancreatitis and tuberculosis. Some benign pancreatic lesions such as chronic lymphoplasmacytic pancreatitis and autoimmune pancreatitis may mimic malignant mass with increased SUVmax (25–28). Researches have been reported that selective use of delayed imaging (usually 2–3 h after injection) is beneficial for differentiating between malignant and benign lesions in pancreas because of the better target non-target ratio (29–31). Our results supported that SUV of malignancies tend to increase over time (or RI > 0) with a high sensitivity of 93.3%, while relatively large variance was observed in benign cases (41.2% increased, 58.8% maintained or decreased). Delayed scan seemed to reduce overlap of SUV obtained in malignant and benign cases and may help improve interpretation confidence especially in benign cases with stable or decreased SUVmax. Future prospective studies are required to better understand the additional value of delayed PET/CT and optimal indication. However, the above parameters were derived from SUV, a semi-quantitative parameter that are known not only as time-dependent values but also as method-dependent ones that can be changed by acquisition conditions, reconstruction methods, region of interest (ROI), plasma glucose level and other factors (32). There are no standard criteria for SUVmax to define an increase in ^18^F-FDG uptake. Thereby, diagnosis should be made after a comprehensive analysis of the images. With the rapid development of computational biology, extracting advanced image texture features from medical images such as PET/CT could provide a wealth of additional information, which may be promising to improve diagnosis and management of patients (33–35).

Serum CA19-9, CECT, and CEMR have their own merits in diagnosis of pancreatic lesions and have been widely used. Serum CA19-9 is the most useful tumor marker for pancreatic cancer but non-specific. One of the disadvantages of this study is the lack of baseline assessment of serum CA19-9 in some cases (especially those with obstructive jaundice or underlying liver conditions), which may explain its relatively lower specificity (69%) than previous reported [80–90% (6)]. Nevertheless, CA19-9 as a more convenient and cheaper blood examination, could significantly improve diagnostic efficiencies when combined with imaging modalities, with PET/CT in particularly. When the results of PET/CT and CA19-9 are consistent (both positive or negative), the likelihood of supporting or excluding malignant pancreatic lesions increases (with high PPV of 100% for serial test and NPV of 95.6% for parallel test). However, we should also be aware of that the improved sensitivities of parallel tests are at the cost of lower specificity and PPV, while serial tests were less sensitive and accurate than PET/CT alone. Given that PET/CT performs better than serum CA19-9, the results of PET/CT may be more informative and reliable when the two results are opposite.

CECT is the standard diagnostic method for pancreatic cancer because of its effectiveness and availability. MRI has superior soft tissue resolution and high sensitivity and is often used as a supplementary modality (6). CT or MRI with intravenous contrast allows precise assessment of the relationship of the primary tumor to the vasculature. Yet both CECT and CEMR are still restricted to morphological portrait of tumor. There are small-scaled individual series that have compared PET/CT with traditional tests in diagnosis of pancreatic lesions, suggesting the incremental diagnostic value of PET/CT (13, 16, 36). A recent prospective study in UK have provided evidence for incremental diagnostic benefits of PET/CT compared to CECT, especially for those who are suspected of having pancreatic cancer on MDCT and planned for surgery (17). Our study showed that PET/CT has similar sensitivity to CECT and CEMR, and significantly higher specificity and accuracy than the other two. PET/CT helped reduce false diagnoses of morphological images, specifically 14.7% (28/191) cases for CECT and 12.2% (10/82) for CEMR. Moreover, combined application with PET/CT can enhance diagnostic efficiencies compared to CECT alone, meanwhile improve specificity of CEMR alone. Our study also showed diagnosis improvement of combination CECT with CEMR but had no significant difference compared with CECT alone. However, the small number of cases in this subgroup (*n* = 45) means that a statistical comparison of the diagnostic tests will have a low power to detect small or moderate effects. These results require further verification by larger sample sizes, and prospective methodology research comparing different diagnosis methods.

A major limitation of the current study was the retrospective nature of data collection from a single center, which may lead to selection and recall bias. However, the large sample size, uniform institutional clinical data system and long-term follow-up strengthen the findings of the study. And we also provided comprehensive comparison to diagnosis efficiency of different methods. Secondly, although various types of pancreatic lesions are covered in our study, the cases of certain lesion type, especially with confirmatory pathology results, were too small to provide meaningful statistical results if analyzed as subgroups. Therefore, our study was mainly focused on the general discrimination between malignant and benign pancreatic lesions of different methods. Although current guidelines (NCCN and ESMO) consider that the role of PET/CT in pancreatic cancer remains unclear, NCCN guidelines suggest that functional PET imaging can be used in high-risk patients to detect extra-pancreatic metastases (6, 8). Further systemic analysis comparing PET/CT with standard diagnostic methods, along with prospective, cost-effective analysis are still required to help to address the issues around the widespread utility of PET/CT. Preliminary data suggest that there was a trend for contrast-enhanced PET/CT to be superior to unenhanced PET/CT in detection and assessment of resectability, providing functional information for whole-body staging for surgical and radiotherapeutic planning (12, 37, 38). However, there was also concern about increasing acquisition time, radiation burden, and contrast-related artifacts that may lead to overestimation of SUV in PET/CT with contrast (39). Some researchers suggested that PET/MR can be done without contrast media in some settings where a contrast-enhanced PET/CT is needed to be diagnostic (40). With the increasing installed base of systems, clinical data will be forthcoming and define more clearly its clinical value in pancreatic cancer.

## Conclusion

^18^F-FDG PET/CT has outstanding value in the diagnosis of pancreatic lesions and performs better than serum CA19-9, CECT, and CEMR, especially in terms of specificity and accuracy. The joint application of PET/CT with other methods could enhance diagnostic efficiency in varying degrees by their advantage complementation.

## Data Availability Statement

The original contributions presented in the study are included in the article/supplementary material, further inquiries can be directed to the corresponding author/s.

## Ethics Statement

The studies involving human participants were reviewed and approved by Institutional Review Board of Union Hospital, Tongji Medical College, Huazhong University of Science and Technology. Written informed consent from the participants' legal guardian/next of kin was not required to participate in this study in accordance with the national legislation and the institutional requirements. Written informed consent was not obtained from the individual(s) for the publication of any potentially identifiable images or data included in this article.

## Author Contributions

SH and HC performed acquisition of data. SH was a major contributor in writing the manuscript. XL substantial contributed to conception and design, drafting the article, and revising it critically for important intellectual content. All authors were involved in the analysis and interpretation of data, read, and approved the final manuscript.

## Funding

This work was supported by the Key Project of National Natural Science Foundation of China (No. 82030052 and 81630049), Hubei Technical Innovation Major Project (No. 2017ACA182), and the Clinical Research Physician Program of Tongji Medical College, Huazhong University of Science and Technology (No. 5001530008).

## Conflict of Interest

The authors declare that the research was conducted in the absence of any commercial or financial relationships that could be construed as a potential conflict of interest.

## Publisher's Note

All claims expressed in this article are solely those of the authors and do not necessarily represent those of their affiliated organizations, or those of the publisher, the editors and the reviewers. Any product that may be evaluated in this article, or claim that may be made by its manufacturer, is not guaranteed or endorsed by the publisher.

## Abbreviations

F-FDG: 2-[^18^F]fluoro-2-deoxyglucose
PET/CT: Positron emission tomography/computed tomography
CA19-9: Carbohydrate antigen 19-9
CECT: Contrast-enhanced computed tomography
CEMR: Contrast-enhanced magnetic resonance imaging
GLUT-1: Glucose transporter-1
SUVmax: Maximum standardized uptake value
RI: Retention index
ROC: Receiver operating characteristic
AUC: Area under curve
PPV: Positive predictive value
NPV: Negative predictive value
ROI: Region of interest.
